# Effect of antenatal education on pregnant women`s knowledge, attitude and preferences of delivery mode

**DOI:** 10.1186/s12884-024-06922-0

**Published:** 2024-11-12

**Authors:** Eman Hassan El-shrqawy, Amina Elnemer, Hanan Mohamed Elsayed

**Affiliations:** 1 Woman’s Health and Midwifery Nursing, Faculty of Nursing, Dakahlia Governorate, Mansoura University, Mansoura City, Egypt; 2 Professor, Woman’s Health and Midwifery Nursing, Faculty of Nursing, Dakahlia Governorate, El Mansoura City, Egypt

**Keywords:** Antenatal education, Knowledge, Attitude, Preferences, Mode of delivery

## Abstract

**Background:**

Childbirth is considered as the happiest action that occurs in any parent's life. Although childbirth is a natural process, the choice of delivery mode is one of the concerns of pregnant women. Consequently, the objective of this study to assess the effect of antenatal education on pregnant women`s knowledge, attitude, and preferences of delivery mode.

**Methods:**

A quasi-experimental research design was utilized to achieve the study utilizing 140 purposive sample of pregnant women selected from antenatal outpatient clinics at the New Obstetrics and Gynecology Hospital in Mansoura, Egypt.

**Results:**

The study findings reported that there was a significant improvement in total scores of the intervention group`s knowledge and attitude toward the modes of delivery, with a highly statistically significant difference (*p* < 0.001) at post-intervention compared to the control group.

**Conclusion:**

Prenatal education sessions were linked to a significantly improved maternal outcome in terms of knowledge, attitude, and preferences for delivery mode (*p* < 0.001).

**Trial registration:**

ClinicalTrials.gov, NCT06561984, registered on August 19, 2024, retrospectively registered.

## Introduction

Childbirth is a unique dynamic process that influences each point of view in women's life. It is considered a comprehensive experience that incorporates physical, emotional, psychological, developmental, social, cultural, and religious aspects [1]. Despite the fact that childbirth is a natural process, mode of delivery choice is considered one of the concerns of pregnant women. Pregnant women can ensure a safe and healthy delivery by receiving appropriate counseling [2].

Delivery mode refers to selecting either a vaginal or cesarean section. Vaginal delivery is the preferred mode of delivery; even about 10% of normal deliveries may be complicated, and cesarean section delivery is recommended to avoid potential morbidities or mortality for the mother or the fetus [3]. On the other hand, the alarming global increase in CS delivery has persisted. Especially, in high- and middle-income countries, CS might be misused [4]. The main causes for the high rate of CS were socioeconomic status, the operation's accessibility and availability, types of private hospitals, and a shortage of medical specialists in public hospitals [5].

Knowledge is considered the main determinant that influences health outcomes and the first line for adopting a healthy attitude. Pregnant women's decisions regarding the preference of delivery mode have been found to be affected by their knowledge and attitudes about different methods of delivery [6, 7]. Previous studies have consistently demonstrated that pregnant women often have poor knowledge about the mode of delivery [8, 9].

Furthermore, different studies revealed that lack of knowledge is responsible for increasing the percentage of women with unindicated CS, leading to the global rise of CS rates [10, 11]. Additionally, evidence reports that pregnant women who are knowledgeable about the modes of delivery can be able to decide the preferred mode of delivery [12].

Antenatal education is a crucial role of midwives when providing antenatal care to pregnant women [13]. Also, prenatal education is a tool that serves expectant mothers in making safe decisions both before and during childbirth, applying skills for choosing the preferred delivery mode, managing their labor pain, and using abilities for postnatal care, infant care, breastfeeding, and motherhood [14].

### Significance of the study

In Egypt, the cesarean delivery rate has been raised dramatically from 52% in 2014 to reached (72%) in 2022. Moreover, Upper Egypt recorded the highest percentage of cesarean birth rates reached (84%) compared to (70.6%) in 2014, and Lower Egypt recorded (76%) compared to (50.2%) in 2014, which suggests that cesarean delivery might be overused or used for inappropriate indications [4]. In addition, Mansoura University Hospital's C-section rate in 2019 was 65.2% [15].

### Aim of the study

The current study aimed to assess the effect of antenatal education on pregnant women`s knowledge, attitude, and preferences of delivery mode.

### Research hypothesis

In order to accomplish the study's aim, the following research hypotheses are developed:

- H1. Pregnant women who receive antenatal education will have a higher level of knowledge and positive attitude regarding the mode of delivery than those in the control group.
- H0. Pregnant women who receive antenatal education will have poor level of knowledge and negative attitude regarding the mode of delivery than those in the control group.
- H1.The percentage of normal delivery among pregnant women in the interventional group will be higher than those in the control group.
- H0. There was no difference between pregnant women in the intervention and control group regarding the selected mode of delivery.

## Method

### Research design

A quasi-experimental research design was utilized to achieve the study's aim. The study adhered to the guidelines provided in the TREND Statement checklist [16]. The study protocol has been officially registered on ClinicalTrials.gov (Identifier code: NCT06561984).

### Study participants

This study employed a purposive sample comprising of pregnant women who attended antenatal follow-up. The inclusion & exclusion criteria were identified with the help of medical practitioner in the antenatal clinic after initial assessment and taking their obstetric history. Out of the initial 214 pregnant women invited to participate, 60 were excluded with 53 not meeting the specified criteria and 7 declined to participate, and 14 were used for piloting purposes. Ultimately, a total of 140 pregnant women were included in the study. To recruit pregnant women in the study, a multifaceted recruitment strategy was implemented. Flyers describing the study’s objectives and procedures were prominently displayed in the antenatal clinics where the target participants were stationed. Additionally, to maximize accessibility and convenience, flyers were strategically placed in the electronic signature areas of the hospital, including admission and discharge locations. Invitations were also shared in WhatsApp groups with the assistance of nurse managers.

### Study setting

This study was carried out at antenatal outpatient clinics of a New Obstetrics and Gynecology Hospital at Mansoura University Hospital, which is a tertiary care teaching hospital affiliated with Mansoura University. This hospital, located in Mansoura City, holds the important role of being the primary teaching hospital for medical education programs in Dakahlia Governorate, Egypt.

The inclusion criteria were 1) Primigravida and pregnant women with a history of one prior elective cesarean section at least two years between pregnancies, 2) Pregnant women who are 18 to 35 years old, and 3) A singleton pregnancy in the third trimester with a normal position. The exclusion criteria were 1) Pregnant women who has any indication of a cesarean section or obstetrical issues that have arisen during the present pregnancy, and 2) Pregnant woman who suffer from psychological illness.

### Sample size calculation

The following formula [17] can be used to determine the sample size based on data from the literature (Mohammed, 2017), taking into account the level of significance of 5% and the power of study of 80%:

$$\text{n}=(2({\text{Z}{\alpha}}/2+{\text{Z}{\beta}})^2\times \text{p}\left(1-\text{p}\right))/(\text{d})^2$$

where, p = pooled the proportion obtained from the prior study; d = predicted difference in proportion of events; Zα/2 = 1.96 (for 5% level of significance) and Zβ = 0.84 (for 80% power of study). Therefore,

$$\text{n}=(2(1.96 + 0.84)^2\times 0.808 (1-0.808))/((0.187)^2)=69.6$$

Therefore, for each group, a sample size of 70 is needed.

### Data collection tools

Three tools were utilized for data collection:

- Tool (1): Structured interviewing questionnaire
  This tool was designed by the researcher after an extensive review of the related national and international literature and was filled by the researcher. It consists of three parts. The first part consisted of socio-demographic characteristics of studied pregnant women such as age, educational level of the mother and husband, residence, occupation, income, and phone number. The second part was about pregnant women`s obstetrical history including gravidity, parity, history of abortion, and inter-pregnancy interval. The last part was about studied pregnant women `s knowledge toward mode of delivery. It was developed by the researcher to assess pregnant women`s knowledge regarding modes of delivery It consists of 24 questions related to normal delivery, cesarean section, and vaginal birth after cesarean definition, advantages, disadvantages, indications and risks of cesarean section [13, 18–20].

### Scoring system for knowledge

Each item of the correct response was scored by ‘1,’ whereas the incorrect one or don’t know was given ‘zero,’ the knowledge total score was calculated by adding the total scores of all sections. Then, classified into three categories: poor level of knowledge < 50% (scoring 1 -21), fair level of knowledge 50 to < 75% (scoring 22–32), and good level of knowledge ≥ 75% (Scoring 33–44) [20]:

- Tool II: Attitude of the studied pregnant women toward mode of delivery
  It was developed by the researcher. Its primary objective was to assess pregnant women attitude toward delivery mode, it consists of two parts:
  - Part I: Attitude of the studied pregnant women toward vaginal delivery:
    It consists of nine questions that cover maternal attitude toward vaginal delivery such as (Childbirth preparation classes will affect the choice of delivery mode, I am terrified of labor pain and frequent vaginal exam and I advise all women to have a vaginal birth, etc.…).
  - Part II: Attitude of the studied pregnant women toward cesarean section:
    It consists of nine questions that cover pregnant woman attitude toward cesarean section such as **(**I prefer a C.S, because it is less painful than S.V.D, I advise all women to have a C.S, If I knew C.S complications, I would never request C.S and cesarean birth is convenient and safe, etc.….).

### Scoring system

Attitude statements were scored on a 3-point Likert scale (1–3). Each statement was scored as follows 3 for (agree), 2 for (sometimes), and 1 for (Disagree) (positive attitude: ≥ 60%, and negative attitude: < 60%) [2, 21].

- Tool III: Studied pregnant women's preference of delivery mode sheet
  The researcher developed this tool after reviewing the relevant literature to assess pregnant women preference of delivery mode. It included two items (the type of delivery mode as normal vaginal delivery or cesarean section and causes for choosing this mode of delivery, including labor pain, the cost, doctor preference, health beliefs regarding mode of delivery, the most common mode of delivery, having previous knowledge toward the mode of delivery, and having knowledge from an educational program, etc.…) [22].

### Tools validity and reliability

The utilized measures underwent a robust translation process from English to Arabic following Beaton guidelines to ensure linguistic equivalence and cultural suitability. The process involved several essential steps to ensure the accuracy and appropriateness of the translated measures for the Arabic-speaking population. These steps encompassed forward translation, expert panel review, back-translation, pre-testing, and cognitive interviewing. Each step was thoroughly executed to maintain the highest level of precision and relevance in the translated questionnaires [23].

With the assistance of experts, the content validity of the study tools was tested. A specialist in medical statistics also examined these tools. The expert's recommendations were followed, and the tools were adjusted. Before the Arabic versions were finalized, the feedback from the validation procedure was used to improve the item phrasing in order to achieve maximum comprehension. Following translation, 14 pregnant women participated in a pilot testing phase of the tools to find any items that needed to be rephrased in order to improve understanding in the particular sociocultural context that was being addressed. The knowledge's internal consistency (Cronbach's alpha) is 0.902, the attitude toward vaginal delivery is 0.898, and the attitude toward cesarean delivery is 0.901.

### Data collection

From the end of May 2023 until the end of December 2023, data was collected. The researcher used journals, articles, and books to review the national and worldwide scientific literature related to the different aspects of the study in order to develop the educational content. The researcher designed a colored instructional booklet in a simple Arabic language after reviewing Arabic and English literatures. Considering women's culture, the educational booklet was designed at an appropriate reading level and in a culturally appropriate style. Photos were added to it to provide further illustration and to aid the women in understanding the material.

With the assistance of experts, three professors with specializations in women's health and midwifery nursing reviewed the educational booklet. The expert's recommendations were followed, and the booklet was modified to achieve maximum comprehension. Prior to the intervention, the researcher used data collection tools to conduct individual, 15–20 min interviews with each woman to evaluate delivery mode preferences, knowledge, and attitude.

### The intervention

Every woman in the intervention group attended four sessions: The researcher divided the women in the study group into seven small groups, each consisting of ten women per week. There were both theoretical and practical components to the training. For both demonstration and re-demonstration, each group attended four scheduled sessions lasting between thirty and forty-five minutes each. First session: The researcher provided an overview of the educational sessions and then received opinions from pregnant women regarding the mode of delivery. The researcher tried to clarify women's misconceptions regarding normal vaginal delivery. At the end of the session, the participants were encouraged to express their opinions and invited to ask questions to be answered. Pregnant women received the instructional booklet from the researcher, who also explained how to utilize it and urged them to exercise the coping mechanisms on a daily basis. The second session focused on cesarean delivery (definition of CS, indications, advantages and disadvantages, risks, and risks of subsequent pregnancy after CS). Instruction was given by lecture, group discussion, and question and answer. The third session focused on vaginal birth after cesarean section (definition, indications, advantages and disadvantages, and risks). The fourth session focused on childbirth planning and postpartum information, including exercises to facilitate normal labor, Kegel exercises, preparing the birth bag, time for going to the hospital, and self-care for the mother and newborn after birth. Periodic email and WhatsApp reminders were aimed at sustaining commitment throughout the educational session period.

At the end of the fourth session, pregnant women`s knowledge, attitude, and preferences questionnaires were collected again (post-intervention evaluation), to assess the effect of the educational sessions on pregnant women`s knowledge, attitude, and preferences toward modes of delivery. Additionally, data were gathered from the control group during a planned prenatal visit at the same time. Pregnant women in the control group received the instructional materials at the conclusion of the educational sessions. Those sessions described in Table 1:

**Table 1:** Description of health educational sessions plan regarding mode of delivery

Components	Description
Language	Arabic
Introduction	**Highlighted on:** The effect of antenatal education on pregnant women`s knowledge, attitude, and preferences of delivery mode
Intended users	Pregnant women who are in the third trimester of pregnancy and aged 18–35 years old with no psychological problem or no indication to cesarean section
Number of sessions	One session per week, every session lasts for (30–45) minutes, 10 pregnant women for each session, for one month
Goal	These educational sessions aim to provide pregnant women with knowledge and a positive attitude to help them in choosing the mode of delivery
Specific objectives	**At the end of these educational sessions, pregnant women will be able to:** - Enumerating about the different modes of delivery (natural birth—cesarean section – V Back) - Identifying the advantages and disadvantages of different modes of delivery - Discussing the risks of modes of delivery and how to prevent them - Discussing how to overcome birth complications - Enumerating the risks of natural birth after cesarean section and the conditions that must be met to achieve it - Identifying the steps that must be followed to facilitate natural birth after cesarean section - Explains how to plan childbirth and decide on the mode of delivery - Discussing self-care for the mother and baby after birth
Educational materials	Learning written material, pictures, videos, and data show
Educational methods	Lecture, group discussion, and demonstration
Methods of Evaluation	Pre and post- test

### Ethical considerations

Ethical approval was obtained from the Faculty of Nursing Research Ethics Committee (Reference No.P.0316). This study adhered to the standards outlined in the Declaration of Helsinki [24]. Written consent was obtained from all study participants after explaining the objectives, procedures, and potential risks. All participants agreed to participate in this study of their own volition using a consent form provided by the Faculty of Nursing Research Ethics Committee. Pregnant women were further informed that their participation in the study was completely optional and that all data collected would be handled with confidence and utilized only to achieve the objectives of the study. Furthermore, participant women have the choice to withdraw from the study at any time, and for any reason.

### Statistical analysis

Version 20.0 of SPSS for Windows was used for all statistical analyses (SPSS, Chicago, IL). Continuous data were presented as mean standard deviation (SD) with a normal distribution. Numbers and percentages were used to express categorical data. The Chi-square test was employed to compare variables with categorical data, or Fisher's exact test if appropriate. With continuous data, correlations between two variables were examined using a correlation coefficient test. The study's questionnaires' internal consistency test, or reliability test, was computed. The cutoff point for statistical significance was *p* < 0.05.

## Results

Table 2 shows that the mean ages of the studied pregnant women in the control and intervention group were 25.3 ± 3.8 & 25.6 ± 4.3, 70% & 72.9% of them were married at the age 20 – 25 years, 58.6% & 60% respectively of them had higher education, and half of the studied pregnant women husbands had higher education, 60% & 64.3% respectively of the control and intervention group monthly income is not enough, and 62.9%, and 70% respectively of them are from rural areas with no statistical differences (*P* > 005).

**Table 2:** Socio-demographic characteristics of the studied pregnant women *N* = 140

Variables	Control group *N* = 70		Intervention group *N* = 70		Significant test	
	**N**	**%**	**N**	**%**	**X**^**2**^	**P**
**-Age (Years)**						
20 – 25	40	57.1	42	60.0		
26 – 30	22	31.4	18	25.7		
31 – 35	8	11.4	10	14.3	0.671	0.715
Mean ± SD	25.3 ± 3.8		25.6 ± 4.3		0.437	0.662
**-Age at Marriage (Years)**						
< 20	21	30.0	19	27.1		
20 – 25	49	70.0	51	72.9	0.140	0.708
Mean ± SD	21.3 ± 2.3		20.9 ± 2.3		0.884	0.378
**-Women's Educational Level**						
Read and write	5	7.1	9	12.9		
Secondary	24	34.3	19	27.1		
University	41	58.6	42	60.0	1.736	0.420
**-Husband`s Educational Level**						
Read and write	7	10.0	9	12.9		
Secondary	28	40.0	26	37.1		
University	35	50.0	35	50.0	0.324	0.850
**-Family Income**						
Not enough	42	60.0	45	64.3		
Enough	28	40.0	25	35.7	0.273	0.601
**-Residence**						
Rural	44	62.9	49	70.0		
Urban	26	37.1	21	30.0	0.801	0.371

**Fig. 1:**
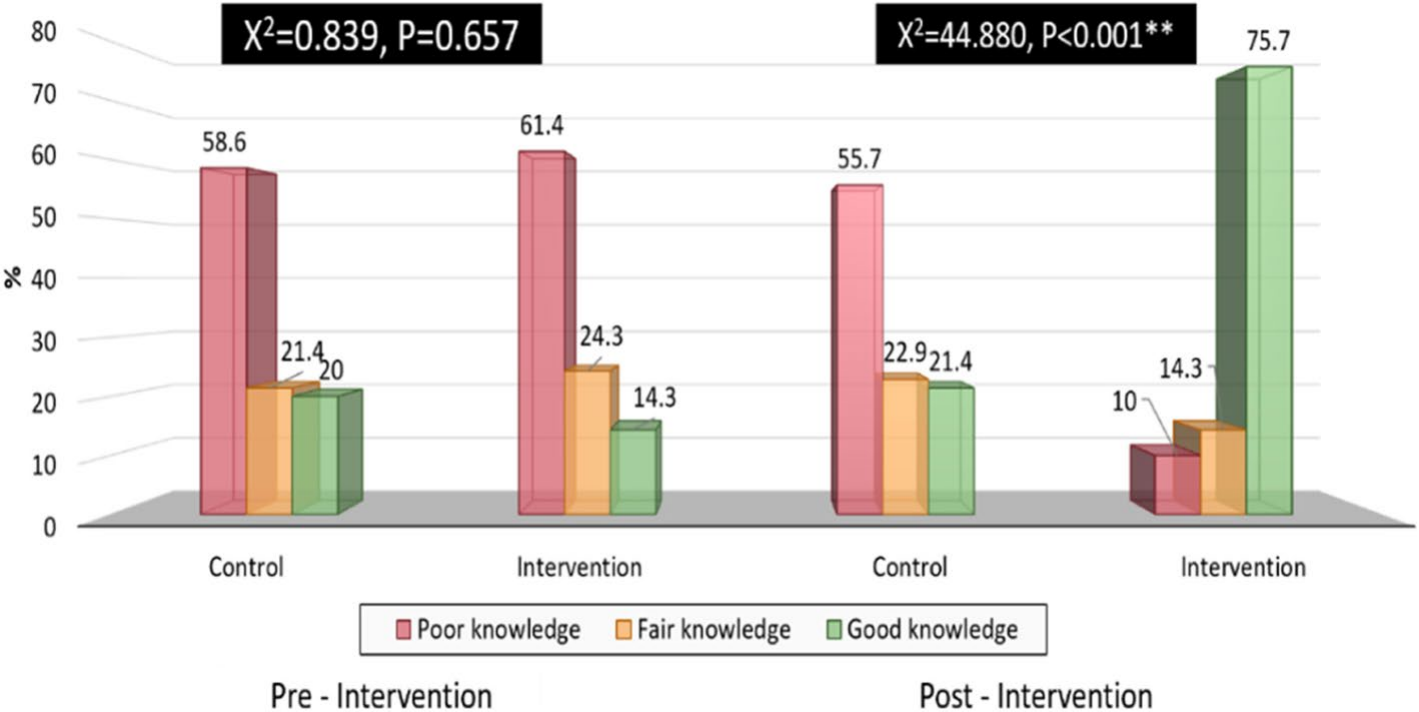
Distribution of total knowledge score among the studied pregnant women pre and post- in

Figure 1 clarifies that (14.3%) of the studied women of the intervention group pre-intervention had good knowledge regarding mode of delivery, which improved to (75.7%) at post-intervention with a highly statistically significant difference (*p* < 0.001).While (20%) of the studied women in the control group pre-intervention had good knowledge regarding mode of delivery, which improved to (21.4% %) at post-intervention with no statistical significant difference (*P* = 0657).

**Fig. 2:**
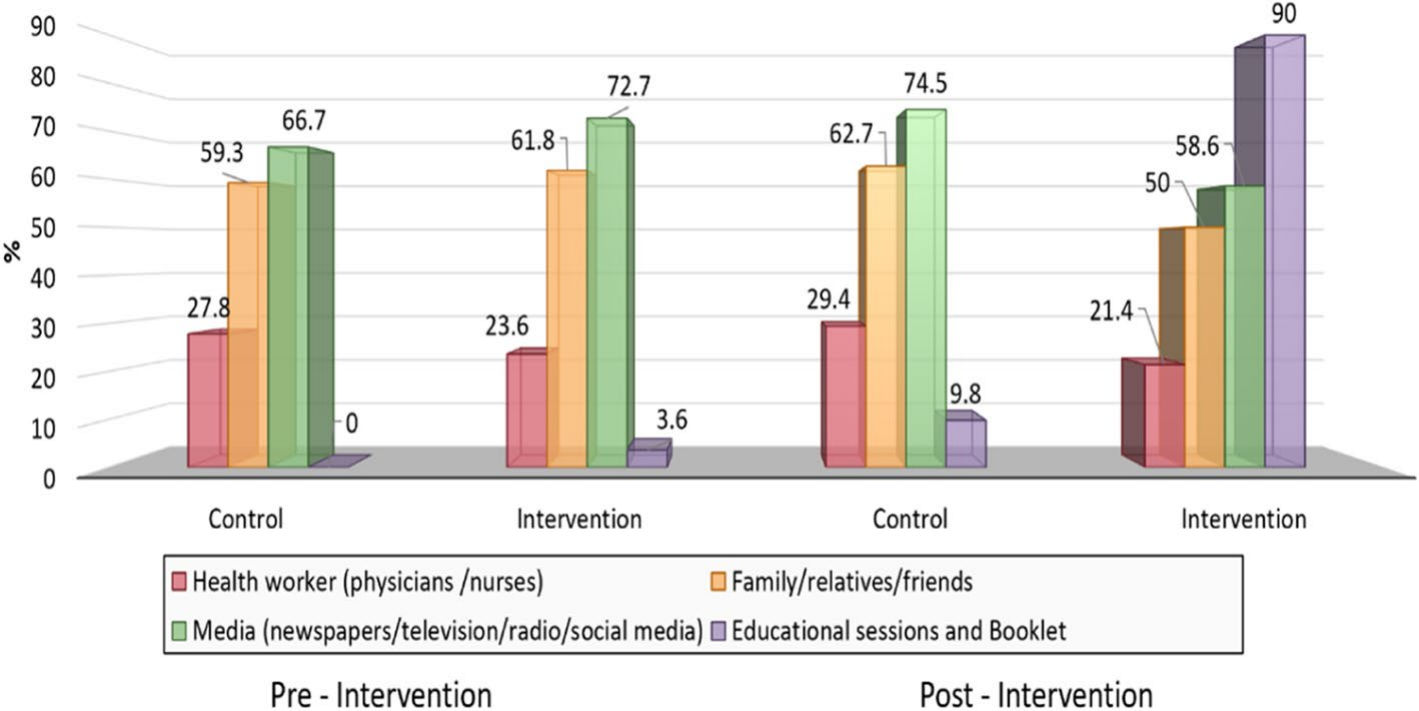
Studied pregnant women`s sources of information regarding delivery modes pre and post- intervention tervention *N* = 140

Figure 2 shows that media (newspapers, television, radio, and social media) was the most prominent source of information for the control & intervention groups at pre-intervention (66.7% and 72.7%, respectively), whereas the intervention group's main sources of information post- intervention were the educational sessions and booklet (90%).

Table 3 shows that there was a highly statistically significant difference between the control &intervention group regarding their total attitude toward vaginal delivery at post- intervention (*p* < 0.001) in which 42.9% of the control group had positive attitude toward vaginal delivery when compared to 75.7% of the intervention group. While there was no statistical difference at pre-intervention (*P* = 0728) in which 37.1% of the control group had positive attitude toward vaginal delivery when compared to 40% of the intervention group.

**Table 3:** Distribution of studied pregnant women`s regarding their total attitude toward vaginal delivery at pre and post-intervention

**Total attitude toward vaginal delivery**	**Control group (*n*** = 70)		**Intervention group (*n*** = 70)		**Significant test**	
	N	%	N	%	X2	P
**Total attitude toward vaginal delivery (Pre – Intervention)**						
Negative Attitude	44	62.9	42	60.0		
Positive Attitude	26	37.1	28	40.0	0.120	0.728
**Total attitude toward vaginal delivery (post – Intervention)**						
Negative Attitude	40	57.1	17	24.3		
Positive Attitude	30	42.9	53	75.7	15.654	< 0.001**

**Fig. 3:**
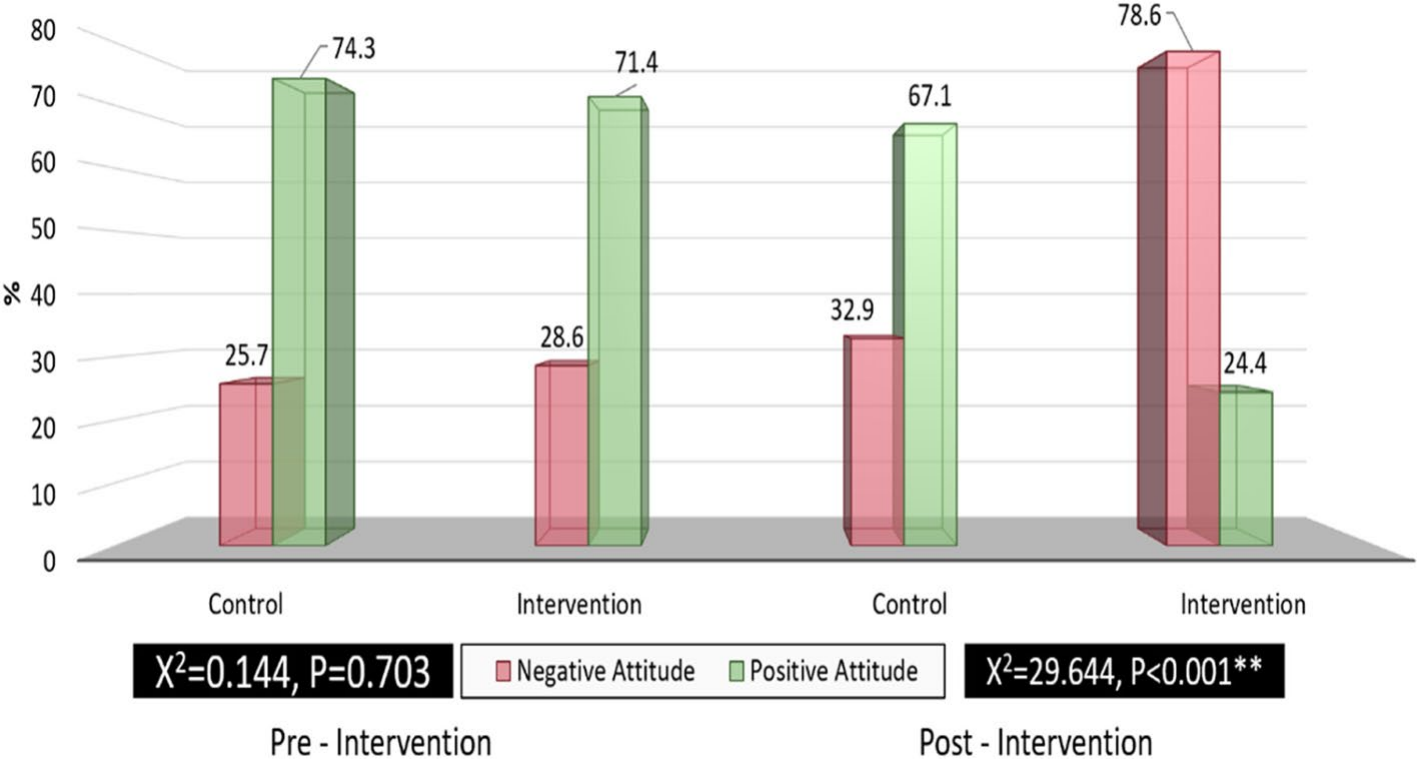
Distribution of studied pregnant women`s regarding their total attitude towards cesarean section pre & post-intervention

Figure 3 shows that there was a highly statistically significant difference between control & intervention group regarding their total attitude toward cesarean section at post-intervention (*p* < 0.001) in which (67.1%) of the control group had positive attitude toward cesarean section compared to (24%) of the intervention group. While, there was no significant difference among control & intervention group at pre-intervention (*P* = 0.703) in which the control group had positive attitude (74.3%) compared to (71.4%) of the intervention group.

Table 4 shows that (30%) of the intervention group had vaginal delivery. While only (10%) of the control group had delivered vaginally at post- intervention.

**Table 4:** Distribution of the studied pregnant women according to their mode of delivery

Mode of delivery of the current pregnancy	Control group (*n* = 70)		Intervention group (*n* = 70)		Significant test	
	**N**	**%**	**N**	**%**	**X**^**2**^	**P**
- Vaginal delivery	7	10.0	19	27.1		
- Emergency caesarean section	32	45.7	38	54.3		
- caesarean section Elective	31	44.3	11	15.7		
- Vaginal delivery after caesarean section	0	0.0	2	2.9	17.586	< 0.001**

## Discussion

This study aimed to evaluate the effect of antenatal education on pregnant women`s knowledge, attitude, and preferences of delivery mode. The aim and hypotheses of the study were supported by the study findings which highlighted that women who acquired good knowledge regarding modes of delivery after attending the antenatal educational sessions experienced positive attitudes toward modes of delivery. As well as study findings revealed that primiparous women had preferred normal vaginal delivery after attending the antenatal educational sessions.

Firstly, the present study evaluated women’s knowledge regarding mode of delivery at the first interview and at the end of four weeks after attending the antenatal educational sessions. The current study results revealed that more than half of the studied women had poor knowledge regarding mode of delivery pre-intervention among intervention & control group where the total score was improved to good knowledge post-intervention for more than three- quarters of the studied women in the intervention group compared to the control group with a highly statistically significant difference. So, the hypothesis that “pregnant women who will attend the educational sessions have good knowledge at post-intervention regarding mode of delivery in comparison to control group”, was supported. The present study findings were supported by the results of [25] who evaluated the effect of birth preparation classes on knowledge, attitude, and preference of delivery method in primiparous women and showed that the mean score of knowledge increased in the intervention group with statistical significance after one month of receiving the antenatal educational sessions toward the modes of delivery. Further research on the impact of an educational program based on a health belief model regarding safe childbirth on the mode of delivery chosen by primigravida was conducted by AlSomali, Bajamal, Esheaba, & AlSomali in 2023, the results showed that more than three-quarters of the intervention group compared to more than one-third of the control group had good overall knowledge following the educational intervention.

Moreover, a recent study conducted by [26], who studied the effect of awareness program regarding motherhood preparation on first-time mothers' knowledge, practices, and emotional status, found a highly statistically significant difference between the mother's knowledge and after awareness program implementation.

The present study showed that, media was the most popular source of information at pre- intervention for the control & intervention groups, whereas educational sessions and booklets were the most popular sources of information at post- intervention. This finding could be supported by Wani, Sultana, Waris, & Rao [27], who conducted a study on sociodemographic factors influencing the choice of the mode of delivery and reported that information from healthcare providers was the most common source of information and was significantly associated with preference for vaginal delivery. On the other hand, a descriptive study was conducted by Ahmed, & Piro [28] who assessed pregnant women's perception and attitude towards the mode of delivery revealed that family, friends, and other women who had similar experiences were the most significant sources of information for pregnant women. Few participants pointed to books and media as a source of information. Moreover, another contradicting study by [29] who reported that more than half of the sample got their knowledge from family and friends.

Concerning the total attitude toward vaginal delivery, the present study findings shows that more than three-quarters of the intervention group had a positive attitude toward vaginal delivery compared with less than half of the control group at post-intervention, with a highly statistically significant difference (*p* < 0.001). The current study's findings are supported by Tarigan [30] who reported that those who attended the educational intervention experienced significantly positive attitudes toward vaginal delivery. Using mobile health in primiparous women: effect on awareness, attitude, and choice of delivery type was the subject of another well-supported study by Moghbeli et al. [31], which found that nearly three-quarters of the intervention group had a positive attitude toward vaginal delivery at post-intervention with a highly statistically significant difference.

Contradictory to this study finding, a study conducted by Yin et al. [32] on the effect of childbirth fear on the delivery mode revealed that more than half of the studied women had negative attitude toward vaginal delivery due to high-level of childbirth fear.

Regarding the total attitude toward cesarean section, the current study finding revealed that more than three- quarter of the intervention group had changed their attitude from positive to negative attitude toward cesarean section at post-intervention with a highly statistically significant difference. This finding was in agreement with Solhi, Hosseini & Kamrani [25] who reported that a higher percentage of the intervention group had a negative attitude toward cesarean section with statistical significance after one month of attending antenatal educational sessions. In contradiction to this finding, [33] conducted a descriptive cross-sectional study on the influence of social media on pregnant women’s stress, attitude and fear toward the choice of delivery mode and reported that more than three-quarters of the studied women had a neutral attitude toward delivery mode. This could be related to the fact that pregnant woman's attitude is affected by many factors, such as fear of pain, humiliation, and support from family and physicians.

Concerning the mode of delivery of the current pregnancy, the current study finding revealed that one third of the intervention group delivered vaginally. While only one tenth of the control group had delivered vaginally. So, the second (H2) hypothesis in the present study "the percentage of normal delivery among women in the interventional group will be higher than those in the control group" was supported. This finding can be explained by elective cesarean section rates can be reduced by eliminating the fear of delivery room, which can be attained through providing adequate information about labor and contractions in the antenatal period. So, it was found that pregnant women who received antenatal education had more vaginal births than the control group.

The current study finding in the line with [34] who studied the effect of birth preparation coaching sessions on women's self-efficacy for coping with labor pains and outcomes who found that childbirth education session attendees had increased rate of normal vaginal birth and a fewer of caesarean deliveries. Furthermore, the present study findings were supported by the results of [35] who evaluated the effect of m Health application based on continuous support and education on fear of childbirth, self-efficacy, and birth mode in primiparous and found that the intervention group had a lower rate of CS than the control group.

## Conclusion

In summary, this study employed a quasi-experimental design (Intervention & control group). The main objective of the study was to examine the effect of antenatal education on pregnant women`s knowledge, attitude, and preferences of delivery mode. Based on the present study findings, it is concluded that there were highly statistically significant improvements in the studied women’s total knowledge and attitude regarding modes of delivery after attending the antenatal educational sessions. The results also highlighted that implementing antenatal educational sessions regarding modes of delivery is effective in improving the rate of normal vaginal delivery for pregnant women.

## Recommendation

In the light of the study findings, the following is recommended:

- Designing and applying antenatal education sessions as an essential component of standard antenatal care in all antenatal clinics at public & private hospitals.
- Train the health care providers at the antenatal care clinics and the obstetric hospitals on the implementation of antenatal sessions for primigravida women regarding modes of delivery because she can change women’s knowledge and attitude.
- Husbands should be encouraged to participate in the antenatal sessions to support them and alleviate their fear and anxiety.
- Further research is needed to assess the long-term effects of such program.
- Develop a healthcare information system linking the healthcare providers at the antenatal care clinics or the obstetric hospitals to encourage pregnant women to adhere to the recommended self-care and to continue the follow-up.

### Study limitation

The findings might be difficult to generalize to all mothers in Egypt because this study was performed in only single health care facility. In addition, the study is limited by a sample size. Future research should aim to include more diverse and large samples and encompasses multiple health care facilities to validate and extent the results. Despite the limitations of the study, we hope this study can add knowledge for mothers and healthcare practitioners. It will be a baseline for future research on the Egyptian population.

## Data Availability

The quantitative datasets used and analyzed in this study are available from the corresponding author on reasonable request.

## Abbreviations

C.S: Cesarean section
SVD: Spontaneous Vaginal Delivery
CAMPAS: Central Agency for Public Mobilization and Statistics
